# Recent Progress on Graphene-Functionalized Metasurfaces for Tunable Phase and Polarization Control

**DOI:** 10.3390/nano9030398

**Published:** 2019-03-08

**Authors:** Jierong Cheng, Fei Fan, Shengjiang Chang

**Affiliations:** 1 Institute of Modern Optics, Nankai University, Tianjin 300350, China; chengjr@nankai.edu.cn (J.C.); 014054@nankai.edu.cn (F.F.); 2 Tianjin Key Laboratory of Optoelectronic Sensor and Sensing Network Technology, Tianjin 300350, China

**Keywords:** graphene, metasurface, phase shift, polarization, wavefront shaping, tunability

## Abstract

The combination of graphene and a metasurface holds great promise for dynamic manipulation of the electromagnetic wave from low terahertz to mid-infrared. The optical response of graphene is significantly enhanced by the highly-localized fields in the meta-atoms, and the characteristics of meta-atoms can in turn be modulated in a large dynamic range through electrical doping of graphene. Graphene metasurfaces are initially focused on intensity modulation as modulators and tunable absorbers. In this paper, we review the recent progress of graphene metasurfaces for active control of the phase and the polarization. The related applications involve, but are not limited to lenses with tunable intensity or focal length, dynamic beam scanning, wave plates with tunable frequency, switchable polarizers, and real-time generation of an arbitrary polarization state, all by tuning the gate voltage of graphene. The review is concluded with a discussion of the existing challenges and the personal perspective of future directions.

## 1. Introduction

The electromagnetic response of an optical device, from basic lenses and mirrors to advanced metasurface alternatives, is governed by two factors: material and structure. Beam engineering in conventional optics relies on separate contributions from the material refractive index *n* and the interface profiles d(x,y), leading to bulky size and limited functionality. Driven by the continuous demand on minimization and the progress of nano-fabrication, metasurfaces offer a promising platform for beam transformation over a thin layer of meta-atoms [1,2,3]. Meta-atoms with deep subwavelength structures couple to the light with strong field localization such that the effective material property is modified locally as $n_{eff}\left(x,y\right)$, which is quite different from that of the natural material. With such spatially-designable $n_{eff}\left(x,y\right)$, beam molding is possible over an ultrathin layer of meta-atoms with fixed thickness *d*. Metasurfaces significantly reduce the size of optical devices with comparable or even unavailable novel functionalities, such as a metalens with close-to-unity numerical aperture and a planar profile [4,5,6], polarization-multiplexed holography [7,8,9,10], the synthesis of multiple functions [11,12,13], and coupling of spin-angular momentum [14,15], to name a few.

Actively-tunable or reconfigurable responses are highly desired in practical applications including dynamic wavefront shaping, adaptive optics, and single-pixel detection, which can be facilitated by including functional materials in the metasurfaces with dynamic modulation. Graphene, a single layer of carbon atoms arranged in a honey-comb lattice, is an outstanding candidate with extraordinary electrical and optical properties [16,17], as its Fermi level and the corresponding charge carrier density can be drastically modified by DC gate biasing. In graphene metasurfaces, the material and structure promote each other such that the resonator strengthens the interaction of light with the atomic thin layer, and the tunable material property of graphene in turn modulates the response of meta-atoms. Therefore, the structured graphene or integration of graphene with passive metasurfaces is an effective solution to enhance the dynamic modulation of beam engineering.

Compared to metasurfaces employing other active materials with different tuning mechanisms, such as electrically-tuned liquid crystals [18,19], thermally-modulated phase change materials [20], and mechanically-deformed elastic materials [21,22,23], graphene metasurfaces have several advantages. Graphene as the thinnest active material is naturally compatible with planar metasurfaces and silicon photonics. The electrical tuning speed is very high, from MHz [24,25] to GHz [26], with a large dynamic tuning range or tuning depth. More importantly, the material property of graphene can be electrically modulated in an extremely wide spectrum from sub-terahertz (sub-THz) to near-infrared (near-IR). Interestingly, the conductivity modulation in different sub-bands is quite different, with modulation focused on the real part at sub-THz and on the imaginary part at higher frequencies, leading to various adjustable properties in metadevices.

The development of graphene metasurfaces starts from intensity modulation. In 2011, the intensity modulation of the THz wave in graphene micro-ribbon array was reported [27], followed by several designs as modulators and switches [24,25,28,29,30,31], tunable metasurface absorbers [26,32]. and spatial light modulators [33]. Besides intensity modulation, increasing attention has focused in recent years on the phase modulation for dynamic wavefront shaping and tunable polarization control, with plenty of theoretical designs and several experimental breakthroughs. This paper emphasizes recent development of graphene metasurfaces for active control of phase and polarization, which has not been systematically summarized in related review articles [3,34,35].

The paper is organized as follows. Section 2 introduces the distinct material properties of graphene in different frequency bands with a tunable Fermi level, which lays the physical foundation for graphene-based active metasurfaces and offers different mechanisms of the tunability. Section 3 summarizes the dynamic graphene phase shifters with the target to achieve a large range of dynamic phase response for desired wavefront shaping working in transmission, reflection, in-plane, and out-of-plane manners. Section 4 is focused on the polarization manipulation to achieve tunable polarization selectivity and polarization conversion. Conclusions and personal remarks on the challenges and development directions of graphene metasurfaces are given in Section 5.

## 2. Material Properties of Graphene

The optical response of graphene is governed fundamentally by its surface conductivity. Since the intuitive optical characterization is usually done through the permittivity or the mode index, we summarize in this section the conductivity, the effective permittivity, and the mode index of the surface plasmon (SP) wave supported by graphene, so as to perceive the transformation of optical response with Fermi level and in different frequency bands.

The surface conductivity of graphene can be modeled by the well-known Kubo formula derived with the random phase approximation (RPA) theory [36,37,38].

(1) $$\sigma =\frac{-ie^{2}}{\pi \hbar^{2}\left(\omega +i/\tau \right)}\int_{0}^{\infty }\epsilon \left[\frac{\partial f_{d}\left(\epsilon \right)}{\partial \epsilon }-\frac{\partial f_{d}\left(-\epsilon \right)}{\partial \epsilon }\right]d\epsilon +\frac{ie^{2}\left(\omega +i/\tau \right)}{\pi \hbar^{2}}\int_{0}^{\infty }\frac{f_{d}\left(\epsilon \right)-f_{d}\left(-\epsilon \right)}{{\left(\omega +i/\tau \right)}^{2}-4{\left(\epsilon /\hbar \right)}^{2}}d\epsilon$$

where *e* is the electron charge, ℏ is the reduced Planck constant, and ω is the angular frequency. $f_{d}\left(\epsilon \right)$ is the Fermi–Dirac distribution as $f_{d}\left(\epsilon \right)={\left(e^{\left(\epsilon -E_{F}\right)/k_{B}T}+1\right)}^{-1}$. $k_{B}$ is the Boltzmann constant. *T* is the temperature. $E_{F}$ is the Fermi level. τ is the carrier relaxation time defined by $\tau =\mu E_{F}/\left(ev_{f}^{2}\right)$. $v_{f}=10^{6}$ m/s is the Fermi velocity, and μ is the carrier mobility fluctuating in a large range determined by the fabrication process. μ is around 0.1 m${}^{2}/$Vs in the chemical vapor deposition (CVD) graphene [39], 1 m${}^{2}/$Vs in the mechanically-exfoliated graphene [40], and even 100 m${}^{2}/$Vs in the suspend graphene at low temperature [41]. The carrier density *n* is related to the Fermi level by $n=E_{F}^{2}/\left(\pi \hbar^{2}v_{f}^{2}\right)$. The dynamic modulation of *n* with $E_{F}$ makes graphene either metallic, semiconductive, or dielectric.

The first term in Equation (1) is the intraband contribution, which can be explicitly expressed as [38]:

(2) $$\sigma^{intra}=\frac{2k_{B}Te^{2}}{\pi \hbar^{2}}\ln\left(2\cos h\frac{E_{F}}{2k_{B}T}\right)\frac{i}{\omega +i/\tau }$$

When $E_{F}\gg k_{B}T$, Equation (2) is simplified as the Drude model $\sigma^{intra}=\frac{ie^{2}E_{F}}{\pi \hbar^{2}\left(\omega +i/\tau \right)}$. The second term in Equation (1) originates from the interband contribution and can be approximated, if $E_{F}\gg k_{B}T$, as [38]:

(3) $$\sigma^{inter}=\frac{ie^{2}}{4\pi \hbar }\ln\left(\frac{2E_{F}-\left(\omega +i/\tau \right)}{2E_{F}+\left(\omega +i/\tau \right)}\right)$$

If the thickness *d* is considered, the effective permittivity of graphene relative to the vacuum permittivity is defined as:

(4) $$\epsilon_{r}=\epsilon_{av}+\frac{i\sigma }{\epsilon_{0}\omega d}$$

where $\epsilon_{0}$ is the vacuum permittivity and $\epsilon_{av}$ is the average relative permittivity of the surrounding environment. The mode index of the SP wave supported in the graphene monolayer is derived from the dispersion equation as [42,43]:

(5) $$n_{sp}=\sqrt{\epsilon_{av}-{\left(\frac{2\epsilon_{av}}{\sigma \eta }\right)}^{2}}$$

with η being the wave impedance.

**Figure 1:**
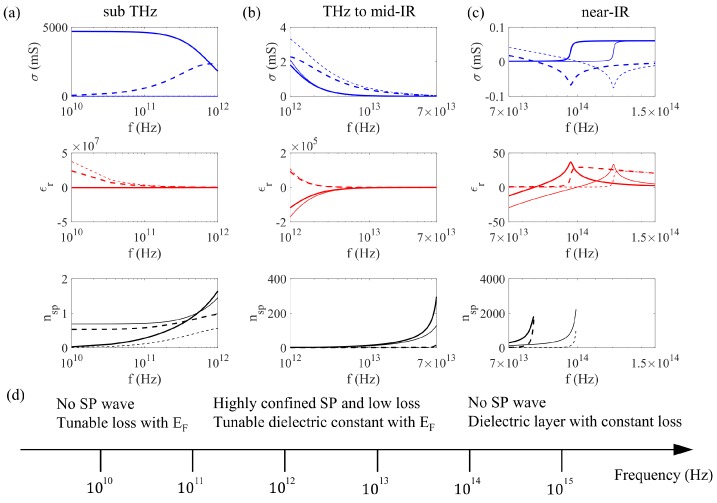
σ (from Equations (2) and (3)), $\epsilon_{r}$ (from Equation (4)), and $n_{sp}$ (from Equation (5)) at sub-THz (**a**), THz to mid-IR (**b**), and near-IR frequencies (**c**). μ=1 m${}^{2}/\mathrm{Vs}$, d=0.35nm, $\epsilon_{av}=1$. The thick lines are for $E_{F}=0.2\;\mathrm{eV}$, and the thin lines are for $E_{F}=0.25\;\mathrm{eV}$. The solid lines are the real parts, and the dash lines are the imaginary parts. The key material properties of graphene are summarized in (**d**) at different frequencies.

In Figure 1, the three parameters above are plotted at different frequencies associated with different types of light–matter interaction. The parameters are specified in the caption. In the sub-THz region (Figure 1a), the real part of the conductivity $\sigma^{{}^{\prime }}$, corresponding to the imaginary part of the permittivity $\epsilon_{r}^{{}^{\prime \prime }}$, is dominant, indicating graphene as a lossy film. The loss is weakly dependent on the frequency, but is very sensitive to the variation of $E_{F}$. Electric gating of the graphene-loaded metallic metasurface in this frequency band is mainly embodied in the amplitude modulation of the local resonances together with a limited range of phase variation due to the transition between underdamped and overdamped phases. Graphene at this frequency is seldom patterned as it does not support the SP wave with $n_{sp}$ less than one.

In the THz to mid-IR band (Figure 1b), the most important feature is the SP wave with very large $n_{sp}^{{}^{\prime }}$ and very small loss. The variation of $E_{F}$ changes $\sigma^{{}^{\prime \prime }}$, $\epsilon_{r}^{{}^{\prime }}$, and $n_{sp}^{{}^{\prime }}$, but only has a small effect on the loss. The metasurface with patterned graphene shows tunable SP resonance frequency together with a large dynamic range of phase variation. Even in the hybrid metasurface where the resonance originates from the metallic antennas, variation of $E_{F}$ shifts the resonance frequency because graphene modulates the dielectric constant of the environment.

When ℏω is comparable to $2E_{F}$ around the step in $\sigma^{{}^{\prime }}$ and $\epsilon_{r}^{{}^{\prime \prime }}$ (Figure 1c), both intraband and interband transitions contribute to the optical response, such that the metasurface with graphene still has tunability by changing the Fermi level. In the near-IR and visible frequencies where $\hbar \omega \gg 2E_{F}$, the interband contribution dominates. $\epsilon_{r}^{{}^{\prime }}$ becomes positive with a constant $\epsilon_{r}^{{}^{\prime \prime }}$. Graphene behaves as a dielectric layer with universal absorption of 2.3% [44].

The variation of key material properties from sub-THz to near-IR is summarized in Figure 1d. It dictates different modulation mechanisms in the graphene-based metasurfaces, which endows the design with diverse tunability with $E_{F}$, such as shifting the operation frequency, modulating the efficiency of wavefront shaping, switching among multiple functionalities, and dynamic polarization conversion, as will be detailed in Section 3 and Section 4.

## 3. Phase Modulation in Graphene Metasurfaces

Metasurfaces have been applied with great success for complicated wavefront shaping because of their compactness and flexibility when compared to conventional bulky devices. Dynamic wavefront shaping is a highly-desirable feature towards practical applications including, but not limited to adaptive optics and satellite communication. Ideally, the phase shifters should cover the complete 2π phase range with high efficiency or even independent control of the amplitude and phase responses to implement arbitrary kinds of wavefront shaping. In reality, it is not easily achievable by simply tuning the optical properties of an atomic layer. The combination of graphene and a metasurface presents an effective solution to enhancing the interaction with increased phase shifting. The constitutive phase shifters in the graphene metasurface array are either graphene micro-/nano-structures with separate biasing or graphene-hybridized metallic/dielectric resonators with a single-location biasing. The former offers the maximum degree of freedom for dynamic wavefront shaping, and the latter is more practical in implementation.

### 3.1. Wavefront Shaping in Transmission

**Figure 2:**
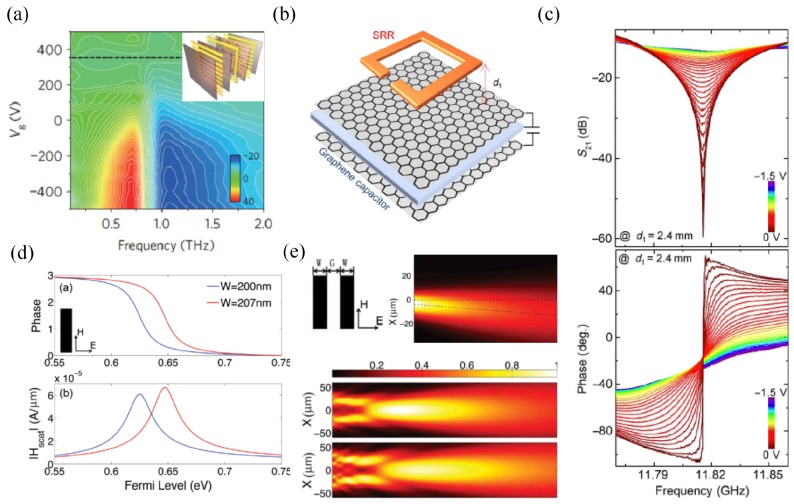
Transmissive graphene phase shifters for dynamic wavefront shaping. (**a**) Transmission phase variation with biasing voltage and frequency in the hexagonal metallic metasurface integrated with graphene. The inset is a schematic of the structure. The dashed line at 350 V corresponds to the charge neutral point. At a fixed frequency 0.65 THz, dynamic phase shift of $32^{{}^{\circ}}$ is observed by tuning the loss of graphene when V${}_{g}$ is from 350 V to −500 V. Reproduced with permission from [28], Copyright Springer Nature, 2012. (**b**) Unit cell of the hybrid metasurface consisting of a split-ring resonator and double-layer graphene capacitor. (**c**) Transmission magnitude and phase spectra at various biasing voltages. The dynamic phase shift is due to the tunable material loss of graphene. Reproduced with permission from [45], Copyright The authors, 2018. (**d**) Phase coverage and scattered magnetic field of uniform graphene ribbons by tuning the Fermi level. The phase coverage is limited to $180^{{}^{\circ}}$. (**e**) Two parallel graphene ribbons are grouped in a unit cell to achieve nearly constant transmission intensity for different phase shifters. An array of such unit cells with individual biasing is simulated for anomalous refraction (top) and focusing with tunable focal length (bottom). Reproduced with permission from [46], Copyright WILEY-VCH Verlag GmbH & Co. KGaA, Weinheim, 2014.

In 2012, Min’s group experimentally demonstrated terahertz switching [28] by attaching graphene to a layer of hexagonal metallic meta-atoms with top and bottom electrodes for static biasing (inset of Figure 2a). Working in the low THz range similar to the case of Figure 1a, the graphene layer acts as the surrounding medium with tunable loss in close proximity to the meta-atoms. The charge carriers accumulated at the edges of the hexagon increasingly leak into the neighboring element through the graphene layer with increased conductivity. Therefore, the resonance becomes weaker and slightly shifted as the biasing voltage is applied from the charge neutral point (CNP). The measured phase response in Figure 2a based on the THz time-domain spectroscopy (THz-TDS) system shows a dynamic phase shift of $32^{{}^{\circ}}$ at 0.65 THz through 850-V voltage variation from the CNP. However, the limited phase coverage is not enough for wavefront shaping. In 2018, more than $90^{{}^{\circ}}$ phase modulation together with the 50-dB amplitude modulation was experimentally demonstrated [45] in a bilayer-graphene-loaded split ring resonator array, as shown in Figure 2b,c.

When graphene itself is patterned as a phase shifter, such as a graphene ribbon or a graphene patch, it only provides a dynamic phase range of $180^{{}^{\circ}}$ around the plasmonic resonance by tuning the Fermi level or the dimension due to the intrinsic Lorentz-shape electric dipole resonance (Figure 2d). In fact, the effect of the material loss and the environment leads to an even smaller phase range [46,47]. Additionally the transmission intensity is very small away from the resonance. To improve the efficiency of such a phase shifter, two graphene nanoribbons are combined in parallel in a unit cell [46]. By individually tuning the Fermi level of each nanoribbon, phase delay varies from 0 to $180^{{}^{\circ}}$ with comparable transmission intensity. Numerical simulations validate an anomalous deflector at IR composed of such elements with tunable beam direction and a flat lens with tunable focal length in Figure 2e. In order to cover the full $360^{{}^{\circ}}$ phase range, a stack of three graphene ribbons with proper dielectric separation was proposed as the metasurface unit cell for efficient beam focusing [48]. The phase shifters achieve a high transmission amplitude above 0.7 with full control over the phase of the transmitted wave when the outer and inner graphene ribbons are independently biased. However, separate gate biasing among elements in three layers is too complicated to implement at the studied mid-IR frequencies.

**Figure 3:**
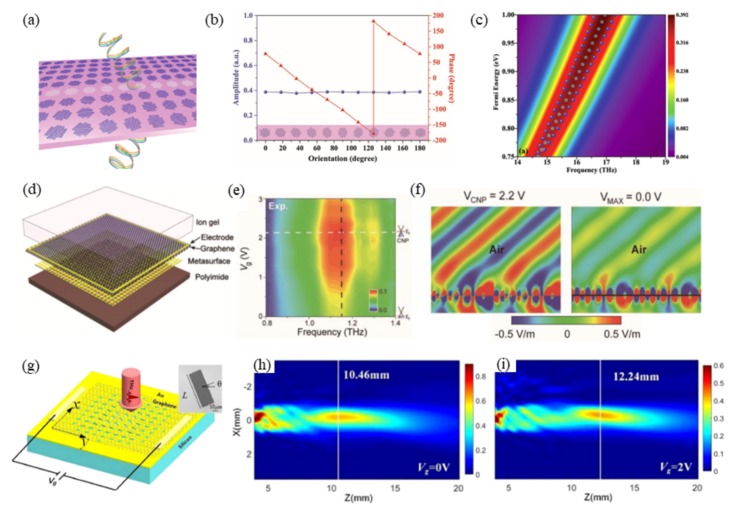
Transmissive active metasurfaces for dynamic wavefront shaping based on the geometric phase. (**a**) Graphene nanocross metasurface with space-variant orientation for deflection of the circularly-polarized beam. (**b**) The transmitted circularly-polarized beam with the opposite handedness of the excitation gains the geometric phase as twice the rotation angle while keeping the transmission amplitude of 0.4 at the optimum frequency. (**c**) Deflection efficiency spectrum when the graphene nanocrosses have different Fermi levels. The large deflection efficiency is well maintained over a wide bandwidth and a large range of the Fermi level. Reproduced with permission from [51], Copyright WILEY-VCH Verlag GmbH & Co. KGaA, Weinheim, 2015. (**d**) Schematic of the active metasurface composed of a single layer of graphene deposited on a U-shaped metallic aperture where the spatial phase function is defined by the orientation of the U-shaped aperture. (**e**) Variation of the efficiency of anomalous refraction with biasing voltage and frequency. (**f**) Electric field distribution at two different gate voltages when the metasurface is designed for anomalous refraction. The metasurface works best at the CNP and shows reduced intensity with additional biasing due to increased loss in graphene. Reproduced with permission from [52], Copyright WILEY-VCH Verlag GmbH & Co. KGaA, Weinheim, 2017. (**g**) Monolayer graphene attached to gold aperture antennas with different orientations for geometric phase response and different lengths for resonance phase response. The resonance phase is tunable by voltage to carefully compensate the variation of the phase function at different frequencies. (**h**,**i**) The phase profile is dynamically tuned to satisfy two parabolic functions at two Fermi levels, leading to different focal lengths with comparable focusing efficiency. Reproduced with permission from [53], Copyright Chinese Laser Press, 2018.

Recently, the geometric phase has been utilized widely in metasurfaces for wavefront shaping by rotating the asymmetric inclusions [49,50]. In Figure 3a, through an array of graphene nanocross resonators with different orientations [51], the transmitted circularly-polarized beam with the opposite handedness of the excitation gains the geometric phase as twice the rotation angle while keeping the transmission amplitude of 0.4 at the optimum frequency. The phase profile for anomalous refraction is solely determined by the orientation of the graphene inclusions irrelevant to the conductivity. Therefore, highly efficient anomalous refraction is maintained over a wide bandwidth from 14.5 THz to 17 THz when the conductivity is dynamically adjusted, as shown in Figure 3b. The increased carrier density of graphene blue shifts the optimum frequency together with a dynamic scan of the beam direction. Although all the graphene elements experience the same voltage, the biasing is complicated due to the separate arrangement. The related study stops at the numerical validation. Until 2018, a similar idea was experimentally demonstrated by Min and Zhang [52] around 1.15 THz with a more feasible structure in Figure 3c. The geometric phase profile is provided by the metallic resonators with proper orientation, on top of which is a graphene monolayer biased at the periphery. For anomalous refraction and focusing, the phase profile does not change with gate voltage, while the intensity does (Figure 3d). Gate-induced increase in the carrier density of graphene results in a stronger absorption of THz waves through the intraband transitions. Therefore, the bending and focusing are most efficient at the CNP and experience an intensity decrease with further biasing (Figure 3e,f). Comparison of Figure 3a,c shows that graphene is a frequency-tunable resonator in the former and loss-tunable medium close to the metallic resonator in the latter, corresponding to the material properties of Figure 1a,b, respectively. Therefore, the optimum working frequency varies with the Fermi level in Figure 3b and stays the same in Figure 3d.

As the geometric phase does not change with the Fermi level, the active tunability of the device is limited to intensity modulation at a fixed frequency. In more scenarios, one wants to tune the focal point or the steering angle dynamically with the maximum possible intensity. For this purpose, Wang’s group proposed a strategy in 2017 to achieve a transmissive metalens with a dynamically-tunable focal length [54]. The experimental demonstration was done very recently [53]. The structure consists of monolayer graphene on top of gold aperture antennas with different orientations and lengths (Figure 3g). The phase profile is determined by both the geometric phase and resonance phase, the latter of which is adjustable by the Fermi level of graphene. With a careful design, the phase profile can be dynamically tuned to satisfy two parabolic functions at two Fermi levels, leading to an electrically-tunable focal length in a large range of 4.45λ in experiments with comparable efficiency, as shown in Figure 3h,i.

### 3.2. Wavefront Shaping in Reflection

When a metallic mirror is introduced to the active phase shifter with a dielectric spacer, the phase modulation range can be significantly enlarged. The whole structure can be considered as an asymmetric Fabry–Perot resonator, where the large phase range is gained from the multi-path propagation between the top and bottom layers. In other words, the electric current circulating between the top metasurface and the bottom plate forms a magnetic resonance with $360^{{}^{\circ}}$ phase variation across the resonance frequency.

**Figure 4:**
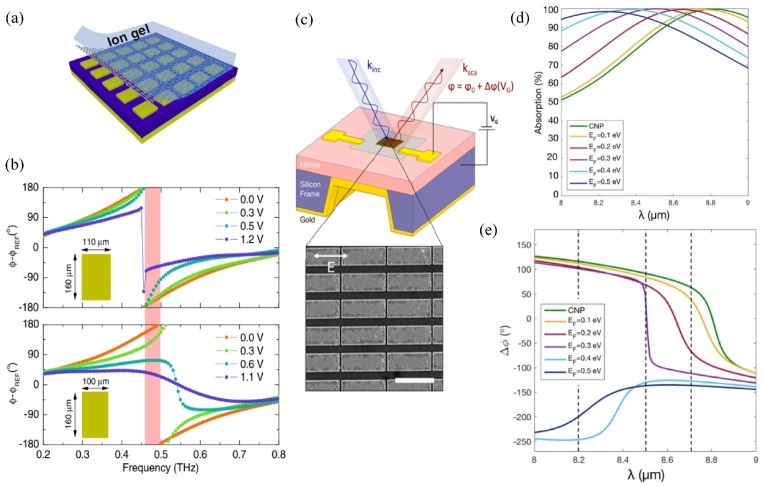
Reflective phase shifters with monolayer graphene for dynamic phase modulation. (**a**) Monolayer graphene is attached to the array of Al patches separated with the bottom Al film with an SU-8 spacer. (**b**) Gate-dependent reflection phase spectra of the combined structure when the Al patches have two sizes. Here, graphene as a lossy surrounding medium in sub-THz frequencies tunes the resonance intensity and phase. In each unit cell, the maximum phase change is $180^{{}^{\circ}}$ around the resonance frequency by tuning the biasing voltage. By utilizing two types of unit cells with different resonance frequencies and working in the shaded frequencies, a $360^{{}^{\circ}}$ phase change is accessible by tuning the biasing voltage. Reproduced with permission from [55], Copyright American Physical Society, 2015. (**c**) Graphene-tuned gold antenna array with back mirror. The zoom-in plot is the SEM image of the gold antennas with nanometer gaps. (**d**,**e**) Tunable absorption (d) and tunable reflection phase (e) for different graphene Fermi levels. The dielectric constant of graphene is sensitively modulated in the mid-IR, leading to a widely-tunable phase range of more than $200^{{}^{\circ}}$ from 8.5μm to 8.7μm. Reproduced with permission from [56], Copyright American Chemical Society, 2017.

In order to access the $360^{{}^{\circ}}$ phase range dynamically at a fixed working frequency, a loss tuning mechanism is required in the top layer to tune the structure through the underdamped-critical coupling-overdamped transition. Tuning the resistive loss of the top layer dictates how far the energy goes through the dielectric spacer and how strong the coupling is with the bottom plate, and is therefore an effective way to tune the phase response. The resistive loss changes the reflection intensity and phase, but does not shift the resonance frequency. One can only access half of the $360^{{}^{\circ}}$ phase range at a fixed frequency with the structure in Figure 4a, where the metallic patches are covered by graphene. The $360^{{}^{\circ}}$ phase range can be covered by two types of metallic patches with shifted resonance frequencies. The working frequency is chosen in the shaded area in Figure 4b between the two resonances [55]. The THz-TDS measurement has demonstrated a maximum phase modulation range of $243^{{}^{\circ}}$ at 0.48 THz, which can be further increased if the carrier scattering loss is reduced in graphene.

The above design works at sub-THz, where graphene is a lossy surrounding medium, as analyzed in Figure 1a. The necessity of two types of unit cells to access $360^{{}^{\circ}}$ phase response originates from the inability of graphene to tune the resonance frequency. Another approach to accessing the full phase range is to work at higher frequencies where graphene directly modulates the resonance frequency with different biasing. In 2017, Atwater’s group demonstrated more than $230^{{}^{\circ}}$ phase modulation using the structure in Figure 4c in mid-IR frequencies [56]. Here, graphene is attached to a uniform array of gold antennas with very small gaps. In mid-IR, graphene shows low loss and a tunable dielectric constant with the carrier density, as discussed in Figure 1b. The resonance frequency shifts with the environmental dielectric constant, resulting in a widely-tunable phase range of more than $200^{{}^{\circ}}$ from 8.5μm to 8.7μm, as shown in Figure 4d,e. Although the measured reflectivity is quite low due to absorption of the SiN${}_{x}$ substrate and the low mobility of graphene charge carriers, the proposed design is one of the most promising designs for dynamic beam steering. Despite the similar configurations in Figure 4a,c, graphene plays different roles in the dynamic adjustment depending on the working frequencies.

**Figure 5:**
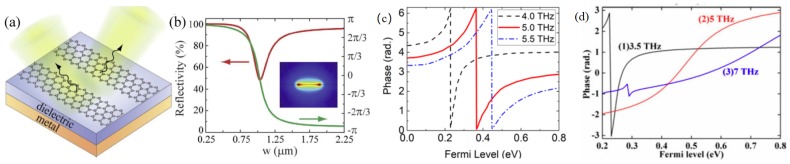
Graphene nanoribbons for reflective wavefront shaping. (**a**) Graphene ribbon element on top of the dielectric/metal substrate. (**b**) Variation of reflectivity and phase with the ribbon width. Reproduced with permission from [47], Copyright The authors, 2015. (**c**) Smooth $360^{{}^{\circ}}$ phase variation is covered by tuning the Fermi level of graphene, and it is shifted with a well-maintained shape from 4 THz to 6 THz. Adapted from [58], Copyright IOP Publishing Ltd., 2017. (**d**) Far away from the optimum frequency, the phase variation is either too sharp or too weak to use. Reproduced with permission from [59], Copyright IEEE, 2018. Wavefront shaping is possible by properly selecting the ribbon width at the fixed Fermi level from (**b**), or by individual gate control over an uniform graphene nanoribbon array following (**c**,**d**).

Alternatively, graphene resonators can replace the graphene–metallic resonator structure in the top layer for reflective phase modulation, as summarized in Figure 5. All of them are targeted at mid-IR beam shaping, where the graphene pattern is a plasmonic resonator with tunable resonance frequency. Among various shapes, the graphene ribbons are the most extensively-studied ones, due to their simplicity and ease of biasing at one end of the structure. Specifically, Figure 5a utilizes the spatially-variant graphene ribbons to achieve desired phase profile at the center frequency and to change the Fermi level for dynamic modulation [47,57]. Figure 5b shows the phase variation with the ribbon width, which serves as the guidance for designing the metasurface array for focusing and steering applications.

When the graphene ribbons are separately biased, more degrees of freedom are gained for controlling the functionality temporally and spatially [58,59]. Figure 5c shows that the smooth $360^{{}^{\circ}}$ phase variation is covered by tuning the Fermi level, and it is well maintained from 4 THz to 6 THz. Beyond this frequency range, the phase variation is either too sharp or too weak to use (Figure 5d). It leads to switching of the functionality by frequency, for example, from an anomalous reflector at 5 THz to a normal reflector at 3.5 THz. With the Fermi level of the graphene ribbon tuned spatially and temporally, a metalens of a large numerical aperture is numerically demonstrated with either a fixed or variable focal point over a wide bandwidth [58]. Completely different functionalities, such as cloaking, illusion, and focusing, can be implemented in one metasurface [60]. In such kinds of active designs, the efficiency is mainly governed by the mobility of graphene carriers. The study shows that the carrier relaxation time τ seldom affects the reflection phase, but mainly controls the local reflectivity. τ is around 1 ps in the designs of Figure 5, leading to an overall efficiency of around 60 to 70%. The efficiency can be improved to 90% if the relaxation time is increased to 5 ps [59].

Similar to the transmission cases, uniform biasing of either monolayer graphene or graphene ribbons is promising for fabrication, but limited to efficiency modulation or on-off switching of a specific function. In contrast, individual biasing of each ribbon is extremely difficult to implement in device integration, although at-will wavefront shaping is theoretically possible in real time with satisfactory efficiency. A compromise solution utilizes both the geometric phase and resonance phase with a similar configuration as Figure 3g, but working in the reflection side with a back mirror [61]. The geometric phase governs the general phase profile, and the resonance phase is tuned by the Fermi level to compensate minor phase variations needed for different focal lengths. However, in both transmission and reflection designs, the lens aperture is limited by the small tuning range of the resonance phase with the Fermi level, leading to an inaccurate focal point as compared to the design.

In addition, the metasurface is endowed with increased power when more complicated graphene elements and the corresponding gate configuration are considered. For example, when two graphene patches are combined in a unit cell in-plane [62] or stacked on top of the substrate with individual gate control [63], the wavefront engineering has been studied with an increased bandwidth. Taking advantage of the highly-confined surface plasmon in graphene, a dual-band focusing lens is proposed by stacking two layers of graphene nanoribbons above the gold reflector with negligible crosstalk [64]. The wavefront can be independently engineered in two distinct frequencies with each layer being responsible for one frequency.

### 3.3. In-Plane Plasmonic Wavefront Shaping and Coupling with Out-of-Plane Propagation

Apart from wavefront shaping in free space, control of the SP waves propagating along the graphene surface holds significant promises towards minimization of the photonic integrated circuits. Compared to the noble metal counterparts, graphene plasmonics show low loss and strong confinement together with the tunability via electrical or chemical doping. As will be shown in the following literature, in-plane and out-of-plane beam shaping focus on applications in the THz to mid-IR frequencies taking advantage of the spatially- and temporally-tunable $n_{sp}$ of the SP wave with the Fermi level following the discussion in Figure 1b.

**Figure 6:**
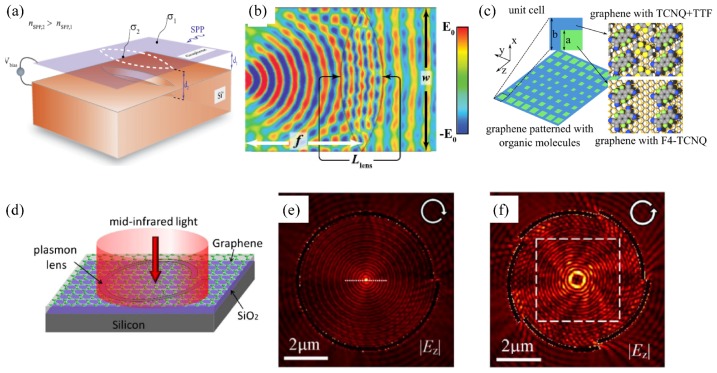
In-plane plasmonic wavefront engineering. (**a**) Monolayer graphene on top of the uneven ground plane made of highly-doped silicon. Different distances between the graphene layer and the ground plane lead to two conductivities and two refractive indices of the SP wave in and out of the lentil area. (**b**) Simulated electric field of the SP wave through the graphene Fourier lens with point source excitation. Reproduce with permission from [66], Copyright Aptara Inc., 2012. (**c**) Schematic representation of the graphene layer patterned with two types of organic molecules (TCNQ + TTF and F4 - TCNQ) to achieve an inhomogeneous conductivity profile through charge transfer. Reproduced with permission from [43], Copyright American Chemical Society, 2014. (**d**) Schematic of the spiral-shaped graphene vacancy for excitation and focusing of the SP wave. (**e**) Superfocusing using a spiral-shaped graphene vacancy together with a circularly-polarized incident beam. (**f**) Generation of plasmonic vortex beam with five segmented graphene vacancies. All the in-plane wavefront shaping is achieved in a notable subwavelength area and beyond the diffraction limit considering the strong localization of the graphene plasmon. Reproduced with permission from [68], Copyright Optical Society of America, 2014.

Fundamentally, the plasmonic wavefront is manipulated via a nonuniform conductivity pattern along graphene. Several ways are proposed to achieve such a conductivity pattern [65]. Figure 6a uses the uneven ground plane to create different distances between the flat graphene layer and the highly-doped silicon ground plane [66]. When a fixed biasing voltage is applied, the electric fields experienced by the graphene inside and outside of the double-convex region are different, which in turn produces an inhomogeneous conductivity pattern for spatial Fourier transformation in Figure 6b. With a proper design of the uneven ground plane, the focal length is even variable with the voltage [67]. Chemical doping, though without dynamic control, is another popular way to change the optical properties of graphene. Figure 6c shows that the adsorption of proper molecules on graphene leads to chemical doping through charge transfer. By patterning two types of organic molecules on graphene, a plasmonic metasurface can be realized with any gradient effective refractive index profile to manipulate SP beams as desired [43]. A multiscale theoretical approach combining the first-principles electronic structure calculations and finite-difference time-domain simulations is developed to reconcile the band structure modification by the molecules and the mesoscopic effect on the SP wave propagation. The designed plasmonic Luneburg lens and SELFOC lens are in a notably subwavelength size of around one tenth of the free space wavelength. By creating vacancies with the designed shape in the graphene layer (Figure 6d), this enables excitation of the SP wave from free-space illumination and manipulation of the SP wavefront [68]. Figure 6e,f shows the plasmonic superfocusing and plasmonic vortex beam generation with a well-designed vacancy geometry. The size of the focusing hotspot is far below the diffraction limit considering the strong plasmonic localization in graphene.

**Figure 7:**
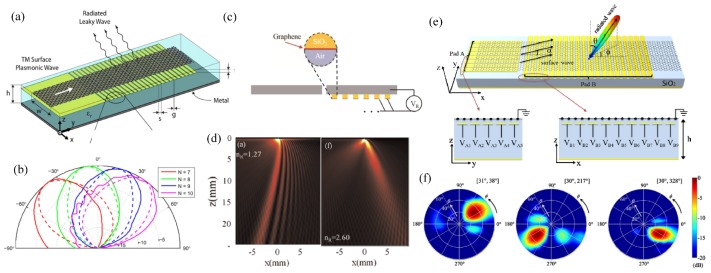
Leaky waves from graphene plasmonic structures with a dynamically-steerable direction. (**a**) A graphene sheet on the back-metalized substrate with the isolated poly-silicon gating pads for space-dependent DC biasing. The surface reactance is sinusoidally modulated with adequate biasing voltage to each pad. The periodic modulation offers effective momentum to transfer the surface wave into free-space radiation. (**b**) The radiation direction of the leaky wave is dynamically shifted when different numbers of pads are contained in a modulation period. Reproduced with permission from [72], Copyright IEEE, 2014. (**c**) The silica-graphene grating with the silver substrate and a slit for THz beam scanning. All the graphene ribbons are biased with the same voltage. The leaky beam direction is determined and tuned by the effective refractive index of the SP wave in graphene. (**d**) The near-field plot of the radiation with different biasing voltages applied. Biasing voltage is 256.5 mV (left) and 53.2 mV (right). The refractive index of the SP wave is 1.27 and 2.60, resulting in the radiation towards $13^{{}^{\circ}}$ and $-18^{{}^{\circ}}$, respectively. Reproduced with permission from [73], Copyright Elsevier B.V., 2015. (**e**) Graphene leaky wave antenna for two-dimensional beam scanning with the simplified two groups of gating pads. One group on the left offering monotonic impedance variation along the y direction mainly determines the radiation in the azimuthal plane, and the other provides sinusoidal impedance modulation along the x direction to decide the elevation angle of the radiation. (**f**) Radiation pattern in different directions via simulation by simply changing the two groups of biasing voltages. Reproduced with permission from [74], Copyright Optical Society of America, 2016.

On the other hand, the strong mismatch of the wave vectors in the plasmonic wave and free space wave causes a great obstacle in exciting and leaking the SP waves in graphene. Different mechanisms have been reported to excite the strongly-localized SP waves in graphene through diffraction gratings [69], sharp tips [70,71], and vacancies [68]. Similarly, the coupling from the SP wave to the propagation wave is the reverse process, which can be done in the same structure. To further increase the flexibility, coupling the SP wave to the free space beam with steerable directions is a highly-desired feature in many applications including communications, remote sensing, and image scanning, which is not easily available especially in the THz region. In the design of Figure 7a, by densely distributing gate pads underneath the monolayer graphene, the surface reactance can be sinusoidally modulated with adequate biasing voltage to each pad [72]. The surface wave interacts with the sinusoidal modulation to produce the leaky wave radiation. Since the radiation direction is determined by the modulation periodicity, which can be dynamically varied by grouping different numbers of pads in one period, the radiation direction can be steered in real time at a fixed frequency, as calculated in Figure 7b. Alternatively, Figure 7c utilizes the periodic metal–dielectric–graphene plasmonic grating to achieve electrically-controllable THz beam scanning [73]. The graphene gratings are biased with the same voltage, which modulates the effective refractive index of the SP wave supported in graphene, leading to varied radiation directions based on the grating equation. Figure 7d shows the radiated near-field from $13^{{}^{\circ}}$ to $-18^{{}^{\circ}}$ when the biasing voltage is 256.5 mV (left) and 53.2 mV (right).

The above leaky wave scanning is limited to one plane. Scanning in arbitrary directions in the full space is more difficult, as pixel-by-pixel biasing makes the configuration a huge challenge for device integration. Figure 7e is a simplified design to achieve such functionality. The dynamic bean scanning in both elevation and azimuth planes is achieved by applying two groups of one-dimensional biasing pads underneath the graphene sheet [74]. They are orthogonal and decoupled. One group offering monotonic impedance variation along the y direction mainly determines the radiation in the azimuthal plane, while the other provides sinusoidal impedance modulation along the x direction to decide the elevation angle of the radiation. Figure 7f shows examples of the two-dimensional radiation pattern towards different directions with the adequate choice of the voltages in the two groups of gating pads.

## 4. Polarization Modulation in Graphene Metasurfaces

Polarization manipulation, usually achieved by birefringence materials, total internal reflection, optical gratings, and the Faraday effect, is instrumental in a wide range of optical applications, such as telecommunications [75], imaging [76], sensing [77], polarimetry [78], and spectroscopy [79]. As compared to those conventional methods, metasurfaces have shown exceptional capabilities for flexible polarization control in a planar, ultrathin, and integrable manner. Introduction of graphene into the metasurfaces opens an exciting route to dynamically and actively taming the polarization in a desired manner. The basic idea is an anisotropic subwavelength pattern made of graphene or hybrid graphene/metal that supports two plasmonic eigenmodes along two orthogonal polarization directions. Therefore, the polarization control generally utilizes the plasmonic property of graphene in Figure 1b. By altering the relative magnitude and phase delay between the two eigenmodes via geometric design and dynamical modulation of graphene conductivity, several outstanding features are proposed, such as wave plates with tunable working frequency, switching between two polarization states, or variation of the polarization along a continuous path in the Poincare sphere, all without readjusting the geometry of the metasurface. The review of this part is classified by the functionality as quarter-wave plates, half-wave plates, polarizers, and general polarization control.

### 4.1. Graphene-Based Quarter-Wave Plate

**Figure 8:**
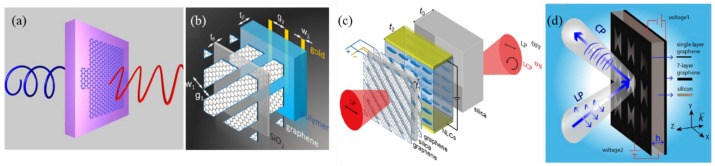
Graphene metasurfaces as active quarter-wave plates. (**a**) Graphene asymmetric nanocross for LP to CP conversion. The $45^{{}^{\circ}}$ LP beam is converted into a CP beam at 7.92μm when the graphene Fermi level is 0.75 eV. The operation frequency blue shifts with the increase of the Fermi level. Reproduced with permission from [80], Copyright Optical Society of America, 2013. (**b**) Hybrid metasurface composed of graphene sandwich grating and gold grating separated by a polymer spacer. The form birefringence in graphene grating and gold grating adds up to offer a constant phase delay of $90^{{}^{\circ}}$ between two eigenmodes over a wide bandwidth. Reproduced with permission from [81], Copyright The authors, 2015. (**c**) Graphene grating sandwich with well-designed distance and in-plane gradient on top of the LC layer. The spatial gradient of the graphene grating increases the form birefringence, and the electrical control of the LC molecule direction leads the QWP to have a dynamic bandwidth of 78%. Reproduced with permission from [82], Copyright The authors, 2018. (**d**) A graphene sheet patterned with butterfly holes and backed by seven layers of graphene with separate biasing for wideband LP to CP conversion. The bottom seven layers of graphene show a tunable reflection phase in order to compensate the difference of the phase delay due to the frequency shift over a wide bandwidth. Reproduced with permission from [83], Copyright The authors, 2017.

The quarter-wave plate (QWP) converts a linear polarization into a circular one, by engineering the two orthogonal eigenmodes with equal amplitude and $90^{{}^{\circ}}$ phase delay. Figure 8a is an asymmetric graphene nanocross with distinct plasmon resonances along the two arms [80]. The $45^{{}^{\circ}}$ linearly-polarized (LP) beam is changed into a circularly-polarized (CP) beam at 7.92μm when the graphene Fermi level is 0.75 eV. The working wavelength is blue-shifted with the further increase of the graphene Fermi level. However, the conversion efficiency is low, and the quarter-wave plate is strictly satisfied at a single frequency. Thus, the bandwidth is very narrow (estimated as 1% from [80]) once a certain biasing voltage is applied. Figure 8b enlarges the bandwidth to 40% due to the interaction of the top graphene grating and the bottom gold grating with opposite phase retardation dispersion [81]. Variation of the carrier density changes the graphene from transparent to conductive, leading to a polarization switch from co-polarized LP to CP with $E_{F}=0$ and 0.5 eV.

The form birefringence of the graphene grating is further enhanced in Figure 8c by introducing a periodic gradient instead of the binary pattern [82]. The increased birefringence shifts the working frequency to a lower value. The bandwidth of the quarter-wave plate relative to the center frequency is therefore increased as compared to the binary design. In order to dynamically move the working frequency in a wide bandwidth, liquid crystal (LC) is integrated into the graphene grating with additional electrical modulation of the birefringence. The theoretical study indicates by electrically controlling the liquid crystal director angle that the dynamic bandwidth of such hybrid quarter-wave plate achieves 78% centered at 1.15 THz. The design in Figure 8d has shown similar dynamic bandwidth by independently biasing the top graphene pattern and the bottom graphene film [83]. The bottom layer composed of seven layers of graphene acts as a reflector with a tunable reflection phase, which is used to compensate the difference of phase delay due to the frequency shift. Furthermore, the efficiency is very high (∼70%) by working in reflection mode.

### 4.2. Graphene-Based Half-Wave Plate

Half-wave plates (HWPs) as the cross-polarization converter require $180^{{}^{\circ}}$ phase retardation and a comparable intensity of the two eigenmodes. Different from the QWP, the HWP is a real challenge through a single layer of anisotropic graphene pattern. As mentioned in Section 3.1, each eigenmode is an electric dipole with $180^{{}^{\circ}}$ or less phase shift across the resonance. Spectral shifting of two dipole resonances from the orthogonal directions of the anisotropic element leads to a phase difference of less than $180^{{}^{\circ}}$. Therefore, the monolayer design working in the transmission mode only rotates the LP wave by a few degrees [84], and almost all the graphene HWPs work in the reflection mode with the anisotropic pattern backed with a metallic mirror at a proper distance. Table 1 summarizes the key features of these HWPs working in THz and mid-IR (all theoretical designs), from which we can generalize the rule of designing graphene HWP to meet the needs of different applications.

The first three designs [85,86,87] in Table 1 share the same L-shaped graphene pattern (G pattern) or the complementary slot. We found that by properly selecting the arm length and width, the performance of the HWP can be narrowband [85], dual-frequency [86], or broadband [87], depending on the spectral separation of the two eigenmodes and their quality factors. Besides the L-shaped design, the graphene layer is patterned into various anisotropic shapes, from which the sinusoidal [88] and ϕ-shaped designs [89] also gain very wide bandwidth. Here, the bandwidth is specific to the static bandwidth when a fixed Fermi level is given to graphene. It is estimated with the polarization conversion ratio (PCR) above 0.5, where PCR is defined as $|R_{cross}{|}^{2}/\left(\right|R_{cross}{|}^{2}+|R_{co}{|}^{2})$. $R_{cross}$ and $R_{co}$ are the reflection coefficients of the cross- and co-polarized beams. The maximum PCR in all cases is close to one, indicating a complete polarization conversion. The carrier relaxation time τ used in the modelings varies from 0.5 ps to 1 ps, and it should be at least larger than 0.02 ps to maintain the polarization conversion effect [90]. The Fermi level is relatively high, close to 1 eV in most cases. An exception is [91], where the graphene Fermi level is 0 eV. The birefringence in this study originates from two layers: the I-shaped metallic resonator and the graphene ribbon with orthogonal orientations, leading to a broad operation bandwidth of around 96%. Although all the designs show very high PCR above 90%, this does not account for the absorption. The peak efficiency listed in Table 1 is the maximum value of $|R_{cross}{|}^{2}$, which indicates the net power into the cross-polarization. Due to the different relaxation times and different intensities of the field localization, the net efficiency varies from 50 to 90%. Still, these HWPs are efficient due to the blocking of the transmission channel.

The lattice size is apparently one or two orders of magnitude smaller than the vacuum wavelength taking advantage of the strong localization of graphene plasmons. As the localization increases with frequency, the lattice size is smaller in higher frequency designs. Due to such a small feature size, all the proposed designs are very robust to the variation of the incidence angle up to $40^{{}^{\circ}}$ or more [88,90,93,94]. This is the merit of the graphene HWP compared to the metallic counterpart besides electrical tunability. Interestingly, the thickness of the dielectric spacer layer separating the graphene pattern and the back mirror is always around 0.25λ if the thickness means the single optical path taking the refractive index of the dielectric into account. The round trip through the dielectric with the $180^{{}^{\circ}}$ phase delay upon back reflection enables the enhancement of interference in the reflection side.

Finally, the dynamic tunability of the graphene-based HWP through the electrical control of the Fermi level is discussed in the last column of Table 1. Generally, an increase of E${}_{F}$ leads to reduced loss and stronger plasmonic resonance with blue-shifted resonance frequency and thus helps increase the PCR. Accordingly, a decrease of E${}_{F}$ causes lower PCR and narrower static bandwidth. The lower limit of the dynamic bandwidth is set by PCR >0.5. In addition, E${}_{F}$ above 1 eV is avoided considering the breakdown of the capacitor and the extraordinarily high biasing voltage, which sets the upper limit of the dynamic bandwidth. The designs in [87,89,94] show a broad dynamic bandwidth around 70∼90%. Table 1 indicates that the design with a wide static bandwidth is more likely to have a wide dynamic bandwidth. Please note that all the data with the ∼ sign are estimated from the plots and without the ∼ sign are the exact ones mentioned in the literature.

### 4.3. Graphene-Based Polarizer

**Figure 9:**
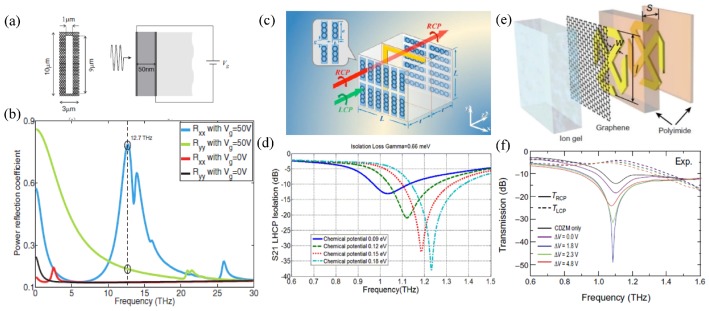
Graphene metasurfaces as active polarizers. (**a**) Rectangular slots in the graphene layer with artificial birefringence and its biasing configuration. (**b**) Different lengths along the x and y directions lead to different resonance frequencies. At the frequency of 12.7 THz, x polarization is on resonance and strongly reflected, while y polarization is off resonance and weakly reflected. The x polarization is filtered upon reflection. Reproduced with permission from [95], Copyright American Physical Society, 2012. (**c**) Two layers of orthogonally-orientated graphene strips sandwiching an L-shaped metallic resonator. The asymmetric L resonator couples to the orthogonal graphene strips, leading the LCP and RCP beams to different resonance frequencies, such that one polarization is blocked by strong resonances and the other goes through with small insertion loss. (**d**) Variation of the LCP transmission with the graphene Fermi level leads to a frequency-tunable polarizer. Reproduced with permisson from [96], Copyright Optical Society of America, 2015. (**e**) Graphene as a loss-tunable material attached to the bilayer of a conjugated double-Z chiral metamaterial for active control of the radiation loss. (**f**) Measured transmission spectra for LCP and RCP waves with different gate voltages. The RCP wave experiences underdamped to overdamped phase transition, while the off-resonance LCP wave experiences negligible modulation. The isolation depth is 45 dB at the critical coupling condition and can be actively controlled via biasing. Reproduced with permission from [99], Copyright The authors, 2017.

In addition to engineering the phase retardation of the two eigenmodes, graphene metasurfaces can be tailored to filter selectively one eigenmode while eliminating the orthogonal one so as to be a polarizer, or in other words a polarization-selective surface. A straightforward example is an anisotropic graphene resonator such as Figure 9a supporting two spectrally-separated resonances along orthogonal directions [95]. By working at a frequency where one polarization is on resonance and the other is off resonance, the on-resonance state is strongly reflected, while reflection of the off-resonance state is fairly weak (Figure 9b). Such polarization selectivity is switched on only when the graphene is doped to support the plasmonic resonance.

Besides the switchable function, graphene endows the polarizer with frequency tunability and the active controllable isolation depth of the polarization states with the elaborate designs in Figure 9c,e, respectively. In Figure 9c, the L-shaped trace is inserted into orthogonal-oriented graphene strips to couple the resonances in the two directions such that the whole structure exhibits the CP selectivity [96]. Left circular polarization (LCP) and right circular polarization (RCP) excitation causes different current distributions in the sandwich element with different resonance frequencies, such that one polarization is blocked by strong resonances and the other goes through with small insertion loss. Figure 9d shows that the operation frequency can be dynamically shifted with the variation of the graphene Fermi level.

The chiral metamaterials have been studied with enhanced circular dichroism (CD) [97] and optical activity (OA) [98]. When the graphene layer is incorporated as a loss-controllable medium into the chiral resonators in Figure 9e, the radiation loss of the resonator is controlled by the voltage [99]. Underdamped to overdamped phase transition for the RCP wave is clearly observed in the experiment, while the off-resonance LCP wave experiences negligible modulation (Figure 9f). Therefore, at the critical coupling condition, a giant CD with a 45-dB isolation is experimentally achieved. Different from the plasmonic feature of patterned graphene in most of the polarization controllers, here the tunable loss of the monolayer graphene is the key to tune the CD originating from the chiral meta-atoms.

### 4.4. General Polarization Control in Graphene Metasurfaces

In general, the output beam through graphene metasurfaces may exhibit elliptical polarization, where the tilt angle and/or the ellipticity can be actively engineered with the variation of the graphene Fermi level. Multiple polarization states are flexibly switchable with proper biasing voltage at high modulation speed in a single structure, which is highly demanding for polarization encoding and multiplexing.

**Figure 10:**
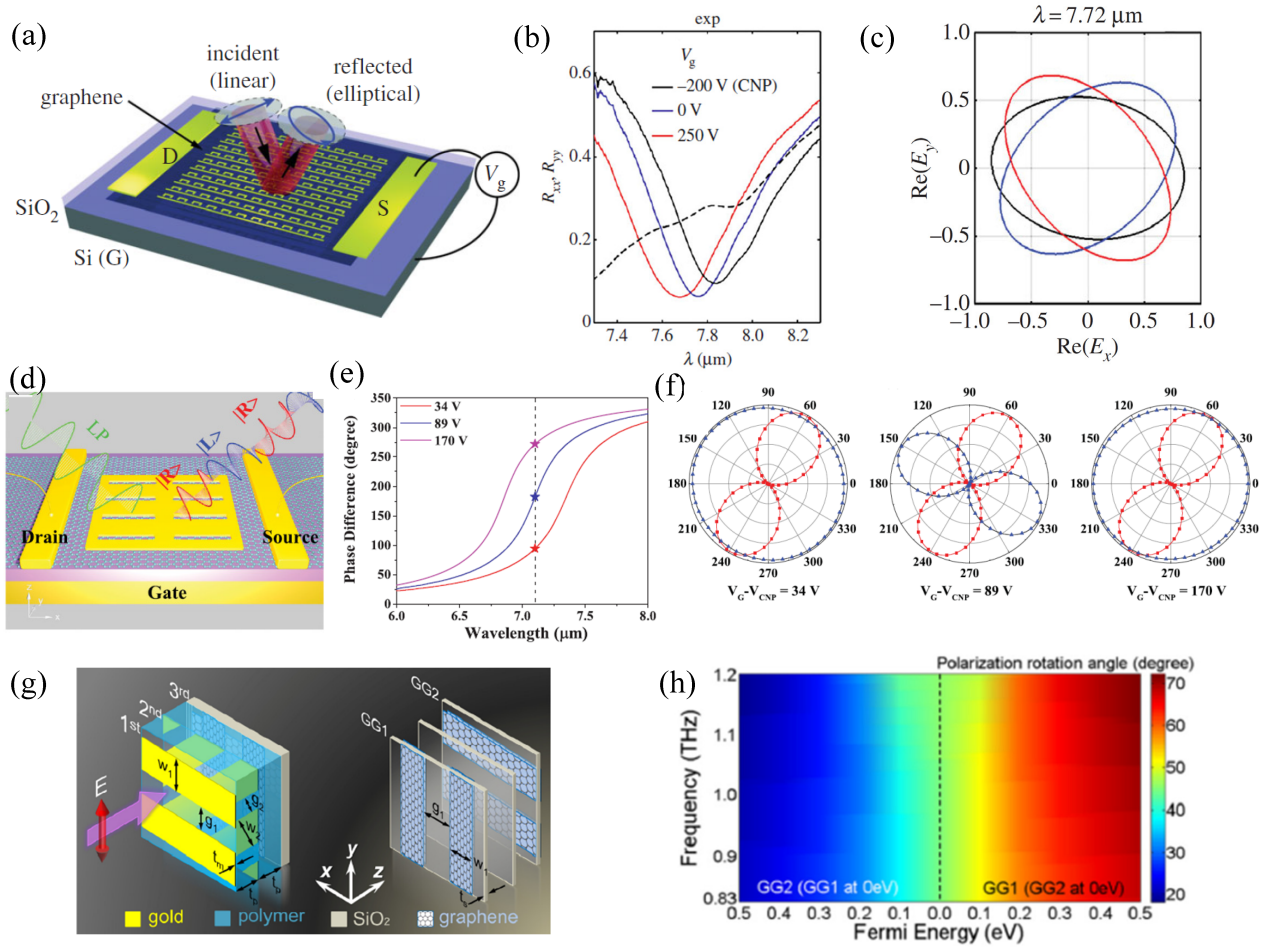
Continuous or multi-state active polarization engineering. (**a**) Graphene integrated with an anisotropic metasurface for linear to elliptical polarization conversion upon reflection. (**b**) Reflection spectra of x- and y-polarized beams with different gate biasing voltages. The design induces a strong Fano resonance only when the polarization is along the bar direction, i.e., the y direction. The Fano resonance dip in y polarization shifts with the voltage, and the x polarization is unaffected. (**c**) The tilt angle is adjustable with constant ellipticity by changing the biasing voltage as −200 V, 0 V, and 250 V at 7.72μm. Reproduced with permission from [100], Copyright The authors, 2017. (**d**) Graphene-loaded slot-shaped metasurface for polarization conversion due to strong resonance along the short edge and weak interaction along the long edge. (**e**) Reflection phase difference between x and y polarization states under different voltages. The phase differences of $90^{{}^{\circ}}$, $180^{{}^{\circ}}$, and $270^{{}^{\circ}}$ are obtained at 7.1 μm with proper voltage applied. (**f**) The $61^{{}^{\circ}}$ linear polarized light is changed to LCP, cross-LP, and RCP when the voltage is 34 V, 89 V, and 170 V relative to the charge neutral point (CNP) for encoding and multiplexing applications. Reproduced with permission from [101], Copyright WILEY-VCH Verlag GmbH & Co. KGaA, Weinheim, 2015. (**g**) Unit cell of a tunable polarization rotator composed of $45^{{}^{\circ}}$-rotated bi-layer metallic gratings and orthogonal bi-layer graphene gratings. The polarization rotation is enhanced by the Fabry–Perot interference between the top and bottom gratings and dynamically changed via modification of the graphene conductivity (**h**) The polarization rotation angle varies continuously from $20^{{}^{\circ}}$ to $45^{{}^{\circ}}$ and $45^{{}^{\circ}}$ to $70^{{}^{\circ}}$ by sequentially tuning the Fermi level of the orthogonal graphene gratings with a high transmission efficiency of above 75% in a wide band between 0.83 and 1.2 THz. Reproduced with permission from [102], Copyright Elsevier Ltd., 2018.

In 2017, Shvets’ group experimentally demonstrated an active graphene metasurface that converts a linear polarization into an elliptical one with controllable tilt angle and ellipticity [100]. The configuration is a C-bar-shaped anisotropic metallic metasurface integrated with a graphene layer (Figure 10a), which induces a strong Fano resonance only when the polarization is along the bar direction (y direction). Therefore, the reflection spectra of the y-polarized beam were well controlled by the voltage due to the intense interaction of the in-plane electric field with graphene, while the x polarization is unaffected, as shown by the measurement results in Figure 10b. At a specific wavelength of 7.72 μm, the tilt angle can be modulated with constant ellipticity when the biasing voltages are −200 V, 0 V, and 250 V in Figure 10c.

Similarly, by utilizing another anisotropic metasurface, namely the rectangle slot array in Figure 10d, the polarization conversion comes from strong resonance along the short edge and weak interaction along the long edge [101]. The voltage variation shifts the resonance frequency, as well as the phase difference between the orthogonal LP modes. Figure 10e shows that the phase differences of $90^{{}^{\circ}}$, $180^{{}^{\circ}}$, and $270^{{}^{\circ}}$ are obtained at 7.1 μm with the corresponding voltage applied, which are used to convert an LP mode into LCP, cross-LP, and RCP, respectively (Figure 10f). The three polarization states can be used for encoding binary data and polarization multiplexing with any desired time-domain sequence. In addition, graphene gratings and metallic gratings are stacked with proper orientation in Figure 10g to rotate the polarization direction of LP beam dynamically from $20^{{}^{\circ}}$ to $70^{{}^{\circ}}$ with a high transmission efficiency of above 75% in a wide band between 0.83 and 1.2 THz [102], taking advantage of the Fabry-Perot interference and the dynamic graphene conductivity. Such controllable optical activity without chiral material holds good prospects in biology and spectroscopy.

## 5. Discussion and Conclusions

If the metasurfaces are classified as two categories: graphene-integrated metallic resonator array and patterned graphene resonator array, most of the experimental validations until now fall into the first case. This is not only because of the relatively mature transfer technique [103] of graphene without further patterning steps, but also due to the simple biasing at a single location. A recent study [104] concluded that the graphene/metal hybrid structure, as compared to the graphene-only structure, has a higher quality factor and extinction ratio, with the performance maintained better during the dynamic modulation. In contrast, patterning and independent biasing of the isolated graphene inclusion remains a big challenge, especially at mid-IR, where the feature size is in the nanometer scale.

The relaxation time of carriers in most of the CVD-grown graphene layer is around 10∼30 fs when transferred to different substrates [28,52,56,100]. We notice that this value is one or two orders higher in most of the theoretical designs, though high mobility of $10^{4}$ cm${}^{2}/$Vs has been reported before [40]. It is predicted that the small scattering time of tens of femtoseconds will introduce strong absorption and significantly reduced the range of phase response [88,89,90]. Therefore, improving the quality of graphene remains a key step to boosting the efficiency of active metasurfaces. The designs robust to the graphene loss or utilizing the lossy feature are more favorable in practice. Other practical considerations include ways to increase the carrier density at low gate voltage by using the ion-gel top gate [105] or the bi-layer graphene capacitor [45].

The development of graphene metasurfaces is generally following the track of metallic metasurfaces. The initially-proposed monolayer metallic metasurfaces usually suffer from poor efficiency and limited phase response range. The introduction of the back mirror enhances the interaction of light with the metallic resonators for efficient wavefront shaping in reflection mode. The geometric phase is then successfully used for wideband phase manipulation, and the layered configuration is helpful to improve the efficiency in transmission mode. All of these progresses have been witnessed in the development of graphene metasurfaces. However, most of the graphene configurations stay at the stage of dynamic phase shifters. To continue following the track of metallic metasurfaces, we envision graphene designs with spatially- and temporally-variant phase profiles for complicated wavefront shaping beyond focusing and bending, such as dynamic hologram imaging and reconfigurable structured beam generation, where the static metallic counterparts have been widely studied.

With the recent trend from plasmonic to dielectric metasurfaces due to programmable electric and magnetic responses, graphene-dielectric hybrid design has become one of the most promising directions for active wave engineering with improved efficiency and increased flexibility. Initial studies include enhanced transmission modulation [106,107], tunable electromagnetically-induced transparency [108], quarter-wave plates [109], and very recently, experimentally-demonstrated tunable absorbers [110].

In summary, the recent progress of graphene metasurfaces is overviewed with emphasis on the active phase and polarization control by relating the basic material property to the tunable device functionality. With the introduction of graphene, the metasurface is vested with tunable operation frequency, controllable efficiency, or switchable multiple functionalities. Additionally, graphene also extends the operation frequency of metallic metasurfaces into the sub-THz regime where metals as perfect electric conductors do not interact much with light, and even the near-IR and visible region where the interband transition dominates the conductivity [111]. Although most of the reviewed studies stay in the theoretical designs and numerical validations, we have noticed that active wavefront shaping, such as anomalous reflection [52] and focusing [53], and electrical tuning of the polarization states [99,100] have been proven by experiments in the past two years. We believe more and more active devices based on graphene will become feasible with the advances of the fabrication technique in the near future, to enrich the optical components and to address technical challenges for the THz and mid-IR technology.

## Figures and Tables

**Table 1:** Comparison of active half-wave plates made of graphene reflective metasurfaces. G, graphene.

Ref.	Structure	Freq.	Static Bandwidth	τ E${}_{F}$	Peak Eff.	Lattice Size	Thickness	Dynamic Tunability
[85]	L-shaped G pattern + back mirror	38.9 THz	∼3%	∼0.9 ps 0.9 eV	∼76%	0.02λ	0.23λ	∼13% dynamic bandwidth with E${}_{F}$ from 0.7 to 0.9 eV
[86]	L-shaped G slot + back mirror	31.4 THz 41.3 THz	∼5% ∼3%	1 ps 0.9 eV	∼80%	0.016λ	0.28λ	∼30% and ∼20% dynamic bandwidth with E${}_{F}$ from 0.7 to 1 eV
[87]	L-shaped G pattern + back mirror	6.65 THz	∼50%	1 ps 0.9 eV	∼80%	0.17λ	0.33λ	∼69% dynamic bandwidth with E${}_{F}$ from 0.7 to 1 eV
[92]	rectangle G holes + back mirror	46.8 THz	∼5%	0.5 ps 1 eV	∼49%	0.03λ	0.25λ	∼32% dynamic bandwidth with E${}_{F}$ from 0.6 to 1 eV
[93]	elliptical G pattern + back mirror	22.5 THz	∼9%	1 ps 0.9 eV	∼83%	0.015λ	0.16λ	∼28% dynamic bandwidth with E${}_{F}$ from 0.6 to 0.9 eV
[91]	I-shaped metallic resonator + G ribbons + back mirror	0.67 THz	∼96%	1 ps 0 eV	∼90%	0.27λ	0.25λ	E${}_{F}$ from 0 to 0.6 eV, polarization varies from cross-LP to ellipse to CP
[90]	H-shaped G holes + back mirror	35.7 THz	11%	0.5 ps 1 eV	∼70%	0.02λ	0.26λ	∼37% dynamic bandwidth with E${}_{F}$ from 0.6 to 1 eV
[94]	Cross G pattern + back mirror	7.7 THz	25.8%	1 ps 1 eV	∼80%	0.05λ	0.25λ	∼77% dynamic bandwidth with E${}_{F}$ from 0.5 to 1 eV
[88]	Sinusoidal G holes + back mirror	1.6 THz	47%	1 ps 0.4 eV	∼60%	0.09λ	0.26λ	Shift of the bandwidth with E${}_{F}$ is small relative to the static bandwidth
[89]	ϕ-shaped G pattern + back mirror	5.98 THz	41.9%	0.6 ps 0.6 eV	∼80%	0.04λ	0.25λ	∼88% dynamic bandwidth with E${}_{F}$ from 0.4 to 1 eV
